# Identification of novel SCD1 inhibitor alleviates nonalcoholic fatty liver disease: critical role of liver-adipose axis

**DOI:** 10.1186/s12964-023-01297-9

**Published:** 2023-09-30

**Authors:** Wei Wang, Yulin Kong, Xia Wang, Zhe Wang, Chunlei Tang, Jinyou Li, Qin Yang, Yong Q. Chen, Shenglong Zhu

**Affiliations:** 1 Jiangnan University Medical Center, Wuxi School of Medicine, Jiangnan University, Wuxi, China; 2 School of Life Sciences and Health Engineering, Jiangnan University, Wuxi, China; 3 Affiliated Hospital of Jiangnan University, Wuxi, China

**Keywords:** Non-alcoholic fatty liver disease, E6446, ATF3, Liver-adipose axis

## Abstract

Due to the complexity and incomplete understanding of the crosstalk between liver and adipose tissue, especially the processes of hepatic lipogenesis and adipogenic differentiation, there are currently no effective drugs for the treatment of nonalcoholic fatty liver disease (NAFLD). Stearoyl-coenzyme A desaturase 1 (SCD1), which is abundantly expressed in liver and adipose tissue, may mediate the cross-talk between liver and adipose tissue. Thus, it is essential to develop specific SCD1 inhibitors that target the liver-adipose axis. Herein, we identified a novel SCD1 inhibitor, E6446, through a high-throughput virtual screen. E6646 significantly inhibited adipogenic differentiation and hepatic lipogenesis via SCD1-ATF3 signaling. The SPR results showed that E6446 had a strong interaction ability with SCD1 (K_D_:4.61 μM). Additionally, E6646 significantly decreased hepatic steatosis, hepatic lipid droplet accumulation and insulin resistance in high-fat diet (HFD)-fed mice. Taken together, our findings not only suggest that E6446 can serve as a new, safe and highly effective anti-NAFLD agent for future clinical use but also provide a molecular basis for the future development of SCD1 inhibitors that inhibit both adipogenic differentiation and hepatic lipogenesis.

Video Abstract

**Supplementary Information:**

The online version contains supplementary material available at 10.1186/s12964-023-01297-9.

## Introduction

Nonalcoholic fatty liver disease (NAFLD) is strongly associated with metabolic syndrome and is caused by the overaccumulation of lipids in the liver due to an increased delivery of fatty acids from adipose tissue [1, 2]. The pathological mechanisms underlying NAFLD are complicated, and there is currently no available clinically approved drug treatment.

The evidence suggests that the dysregulation of hepatic lipogenesis and adipogenic differentiation at the liver-adipose axis plays a key role in the development of NAFLD [3, 4]. Thus, exploring potential modulators that mediate the crosstalk between the liver and adipose tissue may help to identify therapeutic approaches for NAFLD. Enzymes involved in fatty acid metabolism are the most drug-able targets and have been studied extensively [5–7]. Among them, stearoyl-coenzyme A desaturase 1 (SCD1), which is abundantly expressed in liver and adipose tissue, is involved in hepatic lipogenesis and adipogenesis and plays an important role in diverse biological processes [5, 8]. Our previous studies also demonstrated that SCD1 is crucial to respiratory chain impairment, resulting in adipocyte differentiation [9, 10]. Moreover, hepatic or adipose SCD1 deficiency protects against diet or genetic-induced obesity, hepatic steatosis, and insulin-resistance [11, 12]. Therefore, SCD1 may serve as a mediator in the crosstalk between the liver and adipose tissue.

Several small molecule inhibitors targeting SCD1 have been developed for cancer therapy [13, 14]. However, obvious side effects have been observed. Among the therapies for NAFLD that are currently in clinical development, aramchol is a fatty acid-bile acid conjugate that attenuates NAFLD [15]. The therapeutic effects of aramchol may be attributed in part to its targeting of hepatic SCD1 [16, 17]. More effective and more targeted SCD1 inhibitors for use in NAFLD treatment are still lacking. Given the substantial potential for SCD1 to act as a mediator of the crosstalk between the liver and adipose tissue, there is an urgent need to develop SCD1-specific inhibitors that can target the liver-adipose axis.

In the current study, based on molecular docking-based virtual high-throughput screening, we identified an SCD1 inhibitor, E6446. Our results demonstrated that E6446 could significantly suppress SCD1 activity, inhibit hepatic lipogenesis and adipogenic differentiation and improve HFD-induced hepatic steatosis via the target liver-adipose axis in an ATF3 dependent manner. Furthermore, this study also provides a molecular basis for the future development of SCD1 inhibitors with high therapeutic indices and suggests the possibility of investigating the use of established drugs for new purposes, thus supporting immediate drug discovery.

## Materials and methods

### Ethical statement

The animal experiments were approved by Animal Ethics Committee of Jiangnan University (JN No: 20210530c0601230[153]) and were carried out the Guide for Care and Use of Laboratory Animals of the School of Medicine of Jiangnan University.

### Reagents

Minimum Essential Medium α (αMEM; Thermo Fisher Scientific, 12561056), Dulbecco’s modified Eagle medium/nutrient mixture F-12 (DMEM/F12; Thermo Fisher Scientific, 11330032), Fetal bovine serum (VivaCell, Shanghai, China, C04001), rosiglitazone (MedChemExpress, HY-17386), L755507 (MedChemExpress, HY-19334), Quizartinib (MedChemExpress, HY-13001), Barasertib (MedChemExpress, HY-10126), Telaglenastat (MedChemExpress, HY-12248), E6446 (MedChemExpress, HY-12756A), trypsin (Sangon Biotech, A100458), cell culture plate (NEST Biotechnology, 703001), cell culture dish (NEST Biotechnology, 704001), Oil Red O (Sangon Biotech, A600395), jetPRIME transfection reagent (Polyplus-transfection, 114–15), MolPure® Endo-free Plasmid Maxi Kit (Yeasen Biotechnology, 19021ES70), Hieff UNICON qPCR SYBR green master mix (Yeasen Biotechnology, 11198ES), polyvinylidene fluoride (PVDF) membranes (Millipore, ISEQ00010), HiScript III first-strand cDNA synthesis kit (Vazyme Biotech, R312-02), second-strand cDNA synthesis kit (Beyotime Biotechnology, D7172), ITS Media Supplement (Beyotime Biotechnology, C0341), radioimmunoprecipitation assay buffer (Beyotime Biotechnology, P0013B), FastPure Cell/Tissue Total RNA Isolation Kit V2 (Vazyme Biotech, RC112), Enhanced chemiluminescent reagent (ECL) (Millipore, WBKLS0500), HiFi PCR mix for next-generation sequencing (NGS) (CWBIO, CW2648), anti-β-actin (ABclonal, AC026), anti-SCD1 (ABclonal, A16429), HRP goat anti-rabbit IgG (ABclonal, AS014).

### Experimental animals

Male C57BL/6J mice (8 weeks old, weighing 21 ± 2 g) were purchased from GemPharmatech Co., Ltd. (Nanjing, China), and housed at room temperature, 12-h light–dark cycle, with free access to food and water. The mice were divided into 3 experimental groups: normal control diet group (NCD; chow diet, 10% of calories derived from fat), high-fat diet (HFD, 60% of calories derived from fat, Research Diets, Beijing, China; D12451) + Vehicle group, and HFD + E6446 group. After 12 weeks, mice were three times in a week oral gavage with Vehicle or 20 mg/kg E6446 (MCE, China) for 6 weeks. The body weight was measured weekly during oral gavage experimental phases. Mice were sacrificed through isoflurane inhalation, and then blood, adipose, and liver samples were collected for further analysis.

### Cell culture

Mouse OP9 and mouse AML12 cell were purchased from China Center for Type Culture Collection (Wuhan, China). Mouse OP9 cells were cultured in MEM supplemented with 5% fetal bovine serum (FBS) and 1% penicillin–streptomycin at 37 °C and 5% CO2. Mouse AML12 cells were cultured in Dulbecco’s modified Eagle medium (DMEM)/nutrient mixture F-12 supplemented with 5% fetal bovine serum (FBS) and 1% penicillin–streptomycin at 37 °C and 5% CO2. The medium was replaced every 3 days if not otherwise stated. To induce adipogenic differentiation, 100% confluent OP9 preadipocytes were stimulated with 1 μM rosiglitazone in DMEM containing 5% FBS for 15 days [9, 10, 18]. Adipogenic differentiation was induced by 1 μM rosiglitazone in DMEM containing 5% FBS with or without 10 μM E6446 for 15 days when OP9 cells grown to 90% confluence. To establish hepatic steatosis model, mouse AML12 cells were stimulated with palmitic acid (PA; 0.125 mM) and oleic acid (OA; 0.25 mM) with or without 10 μM E6446 at the indicated concentrations for 48 h.

### Small interfering RNA (siRNA) transfection or plasmid transfection

Cells were grown to 50–70% confluence and then transfected with 50 nM siRNA or negative control (NC) siRNA 50 nM using jetPRIME transfection reagent according to the manufacturer’s protocol. The siRNA sequences were mouse ATF3: CAGAAUAAACACCUCUGCCAU; mouse SCD1: AGUUUCUAAGGCUACUGUCUUTT. Universal negative control siRNA (GenePharma, A06001) was used as a control. pcDNA3.1-SCD1 was used for SCD1 over-expression in OP9 and AML12 cells, and the pcDNA3.1 empty vector was used as a control.

### Virtual screening based on the structure using libdock

The X-ray crystal structure of SCD1 [Protein Data Bank (PDB) code 4ZYO] was used for the docking studies. The small molecule database L3400, which contained 4121 molecules, used as the screening library. The LibDock module of Discovery Studio 2018 was used to virtual screening. LibDock is a rigid-based docking module. The active sites of SCD1 were defined using the PDB site records. CHARMM force field and the Smart Minimizer algorithm were performed for ligand minimization. All the docked poses were ranked and grouped, and all compounds were ranked according to the LibDock score.

### Metabolic analysis

Total cholesterol (TC), triglyceride (TG), low-density lipoprotein cholesterol (LDL-c), alanine aminotransferase (ALT), and aspartate aminotransferase (AST) levels in mice serum or cells were measured using commercial kits (Nanjing Jiancheng, China). The liver tissues were homogenized in precooled absolute ethanol lysate (1:9, w/v), then supernatant was taken for later analysis. Liver TC, TG, and LDL-c were analyzed using enzyme kits (Nanjing Jiancheng, China) according to the manufacturer’s instructions. For liver ALT and AST, the precooled saline solution (1:9, w/v) was used to homogenize mice liver tissues, then supernatant was analyzed using enzyme kits (Nanjing Jiancheng, China) according to the manufacturer’s instructions. For glucose tolerance testing (GTT), the mice were fasted for 6 h, and blood glucose level was measured before intraperitoneal injection of 1 g/kg glucose and then 15, 30, 60, 90, 120, or 150 min after injection. Area under the curve (AUC) was used to quantify the GTT results.

### Lactate dehydrogenase (LDH) assay

AML12 or OP9 cells were seeded into 96-well plates and treated with 0, 10, 2, 0.4, 0.08 μM E6446 or A939572 with palmitic acid (PA; 0.125 mM) and oleic acid (OA; 0.25 mM) for 48 h. LDH levels in AML12 cells were measured using commercial kits (Nanjing Jiancheng, China).

### CCK8 assay

AML12 or OP9 cells were seeded into 96-well plates and treated with 0, 10, 2, 0.4, 0.08 μM E6446 or A939572 with palmitic acid (PA; 0.125 mM) and oleic acid (OA; 0.25 mM) for 48 h. Next, 10 μL of CCK8 solution was added and incubated at 37 °C with 5% CO2 for 2 h. The absorbance at 450 nm was measured using a microplate reader.

### Fatty acid methyl ester (FAME) analysis

FAME analysis was performed according to our previous study [9]. Briefly, cell lysates were quantified on a Q Exactive™ GC Orbitrap™ GC–MS/MS (Thermo Scientific) with a Rtx-Wax column (Restek, 12423). Injection port and detector temperatures were maintained at 280 °C. Initially, the column temperature was held at 40 °C for 5 min, then was increased at a rate of 40 °C/min to 120 °C for 5 min, then was raised to 190 °C at 10 °C/min for 5 min, and finally to 230 °C at 5 °C/min for 7 min; the total time spent for all fatty acid peaks was approximately 34 min. In order to identify peak locations, retention times were compared with those of known standards (Sigma Chemical). A reference standard (C15:0) was used to quantify the individual fatty acids. Subsequently, the sample was normalized to total cellular protein.

### cDNA library construction and RNA sequencing (RNA-seq)

RNA-seq was performed according to our previous study [18]. For the purpose of constructing the sequencing libraries, a total of 1 μg RNA was obtained from each sample. We converted the RNA into double-stranded cDNA in accordance with the reverse transcription kit instructions, and then we digested and labeled the double-stranded cDNA using Tn5 transposase. The final step was enrichment PCR using the HiFi PCR Mix for NGS. An Agilent 2100 Bioanalyzer was used to quantify the libraries. Using an Illumina NovaSeq instrument, paired-end sequencing of the library was conducted (sequencing was performed by GENEWIZ Biotech). A STAR program was used to map the reads to the mouse genome (http://code.google.com/p/rna-star/). Genes that showed a 1.5-fold change in expression (*P* < 0.05) were considered significantly differentially expressed. Differentially expressed genes (DEGs) were enriched by Gene Ontology (GO) using Metascape (http://metascape.org) [19].

### Western blot

RIPA buffer was used to lysed the total protein from cells, and western blotting was performed according to the procedures previously described. Briefly, protein samples (50 μg) were separated by 10% sodium dodecyl sulfate (SDS)–polyacrylamide gel electrophoresis and electrically transferred to PVDF membranes. The membrane was blocked with 5% defatted milk for 1 h, and then were washed 3 times with TBST. The membranes incubated with primary antibodies against SCD1 and β-actin at 4 °C overnight. Primary antibodies were detected with anti-rabbit IgG conjugated to horseradish peroxidase (HRP). Immunodetection was visualized using an enhanced chemiluminescent (ECL) reagent (Biotanon Biotechnology, Shanghai, China). Quantification of band intensity was performed using ImageJ software (version 1.53; National Institutes of Health).

### RNA extraction and RT–qPCR assay

Total RNA was extracted as previously described [20]. 1 μg RNA reverse transcribed into cDNA using HiScript III first-strand cDNA synthesis kit. RT–qPCR was performed using Hieff UNICON qPCR SYBR Green Master Mix and Roche LightCycler 480 PCR System (Roche Applied Science, Basel, Switzerland). Relative gene expression was determined using the 2 − ΔΔ method. The primers are described in Table 1.

**Table 1:** Primers for RT–qPCR

Gene	Forward (5’-3’)	Reverse (5’-3’)	Organisms
FABP4	AAGGTGAAGAGCATCATAACCCT	TCACGCCTTTCATAACACATTCC	Mouse
C/EBPα	GCGGGAACGCAACAACATC	GTCACTGGTCAACTCCAGCAC	Mouse
FASN	AGAGATCCCGAGACGCTTCT	GCTTGGTCCTTTGAAGTCGAAGA	Mouse
PPARγ	TCGCTGATGCACTGCCTATG	GAGAGGTCCACAGAGCTGATT	Mouse
β-actin	TGTTACCAACTGGGACGACA	CTGGGTCATCTTTTCACGGT	Mouse
SCD1	TTCTTGCGATACACTCTGGTGC	CGGGATTGAATGTTCTTGTCGT	Mouse
ATF3	GAGGATTTTGCTAACCTGACACC	TTGACGGTAACTGACTCCAGC	Mouse
UCP1	AGCCATCTGCATGGGATCAAA	GGGTCGTCCCTTTCCAAAGTG	Mouse
Prdm16	CCAAGGCAAGGGCGAAGAA	AGTCTGGTGGGATTGGAATGT	Mouse
Pgc1α	TTCATCTGAGTATGGAGTCGCT	GGGGGTGAAACCACTTTTGTAA	Mouse
SCD2	AACACGCAGGCTATGATTCA	TGTCACGAAGTTCCTCAGTTG	Mouse
TLR7	GCACTCTTCGCAGCAACTAA	TCTGTTATCTCCTTCCACCTTGT	Mouse
TLR9	GTCAACCTCAGCCACAACAT	TGCCACACTTCACACCATTAG	Mouse
ID3	CTGTCGGAACGTAGCCTGG	GTGGTTCATGTCGTCCAAGAG	Mouse
Ccn2	CCAAGGACCGCACAGCAGTT	CAGGCAGTTGGCTCGCATCATAG	Mouse

### Oil Red O staining

After washing with phosphate-buffered saline (PBS), the cells or liver sections were fixed in 4% paraformaldehyde for 30 min, followed by three washes with PBS. Cells or liver sections were stained for 15 min with 60% saturated Oil Red O. Finally, 60% isopropanol was used to wash the cells or liver sections, followed by hematoxylin counterstaining. The histological images were acquired with a light microscope (3D Histech, Hungary).

### Histological analysis of liver

Haematoxylin and eosin (H&E) staining of liver tissues was performed as previously reported [21], the histological images were acquired with a light microscope (3D Histech, Hungary).

### Surface plasmon resonance (SPR) analysis

SPR experiments were performed using Biacore X100 (Cytiva, United States). HBS-EP buffer (Cytiva, USA) was used as the working buffer, and the SCD1 recombinant protein was diluted to a final concentration of 20 μg/mL. A mixture of NHS and EDC (1:1, v/v) was injected into the instrument to activate the CM5 sensor. Then, the 20 μg/mL SCD1 recombinant protein was injected to immobilize it onto the CM5 chip through amino coupling. Subsequently, a 1 M ethanolamine hydrochloride (pH 8.5) was injected for 7 min to block and activate the chip surface. E6446 (100, 50, 25, 12.5, 6.25, 3.125 μmol/L) and A939572 (100, 50, 25, 12.5, 6.25, 3.125 μmol/L) were then injected using HBS-EP buffer and passed over the immobilized SCD1 chip sensor surface. The binding kinetics of E6446 and SCD1 were calculated based on the fitted data from the analysis software, with time as the abscissa and response values as the ordinate.

### Statistical analysis

The data are presented as the mean ± standard error of the mean (SEM). All experiments were repeated at least three times. GraphPad Prism 8.0, SPSS 19.0, and R 3.6.0 were used for data analyses. Student’s t-tests and one-way analysis of variance were used to analyze the differences between the groups. *P* < 0.05 was regarded as statistically significant.

## Results

### Virtual screening of the clinical compound library against SCD1

**Fig. 1:**
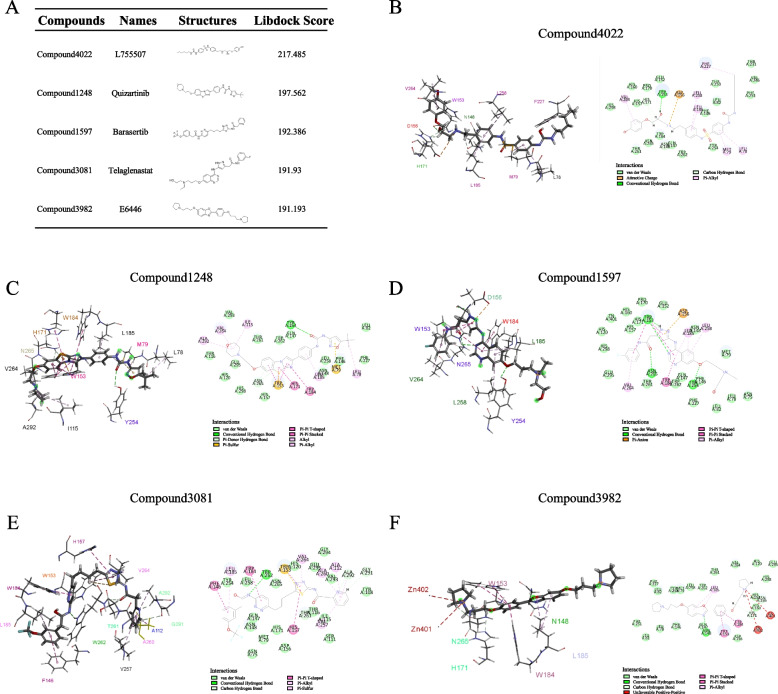
Structure-based virtual screening to identify potential inhibitors. **A** LibDock scores of the top 5 compounds. Compound 4022, Compound 1248, Compound 1597, Compound 3081, and Compound 3982. The intermolecular interactions of Compound 4022 (**B**), Compound 1248 (**C**), Compound 1597 (**D**), Compound 3081 (**E**), and Compound 3982 (**F**) with SCD1

A structure-based virtual screening approach was used to identify potent SCD1 inhibitors. The 3D structure of the SCD1 was retrieved from PDB with the ID 4ZYO. Clinical compounds form the L3400 library were docked to the crystal structure of SCD1 in silico. The results of LibDock indicated that the top 5 compounds with higher scores were selected for further screening and study (Fig. 1A). The top five compounds were Compound4022 (L755507), Compound1248 (Quizartinib), Compound1597 (Barasertib), Compound3081 (Telaglenastat), and Compound3982 (E6446). The interactions between SCD1 and the selective compounds are shown in Fig. 1B-F.

### E6446 inhibits adipogenic differentiation and hepatic lipogenesis

**Fig. 2:**
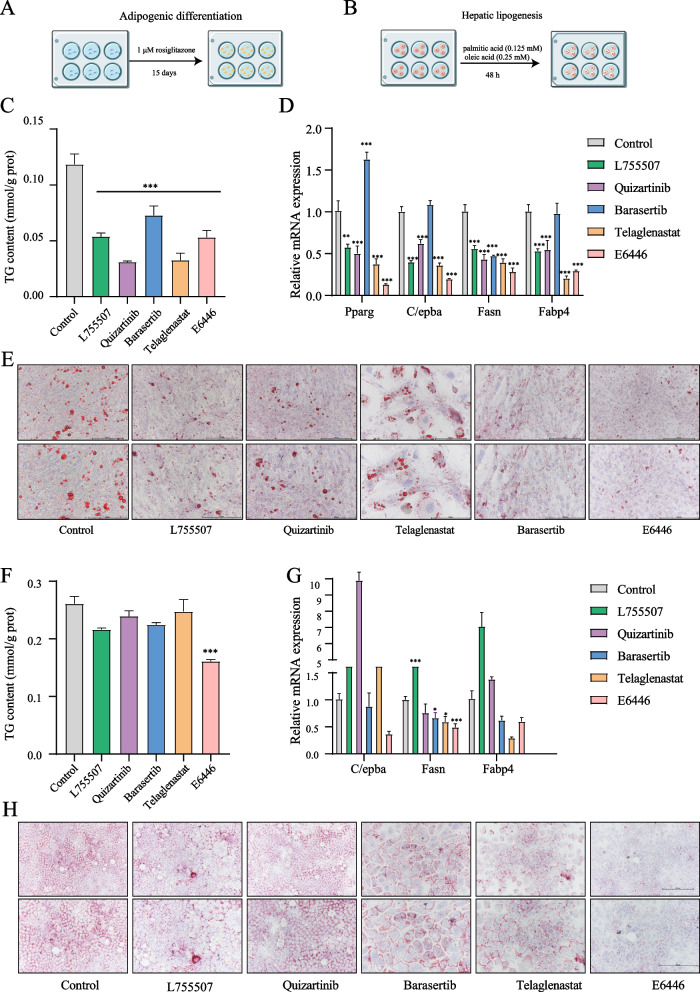
E6446 prevents adipogenic differentiation and hepatic lipogenesis. For adipogenic differentiation, OP9 cells were incubated with 1 μM rosiglitazone for 15 days to induce adipogenic differentiation. Schematic of the adipogenic differentiation (**A**) and hepatic lipogenesis (**B**) strategy. Effect of five potential compounds (10 μM) on the **C** cellular triglyceride (TG) level, **D** mRNA expression of adipogenic differentiation-related genes, and **E** Oil Red O staining (scale bar: 200 μm, 250 μm). For lipogenesis, AML12 cells were treated with a combination of palmitic and oleic acid (PAOA) for 48 h. Effect of five potential compounds (10 μM) on the **F** TG level, **G** mRNA expression of hepatic lipogenesis-related genes, and **H** Oil Red O staining (scale bar: 200 μm, 250 μm). The values presented are the means ± SEMs of three independent experiments. ^*^P < 0.05, ^**^P < 0.01, ^***^P < 0.001 vs. the Control group

To evaluate the pharmacological effects of the top 5 candidate compounds, two classic cell models (adipogenic differentiation and hepatic steatosis models), which model the most critical processes of fatty liver formation, were selected in this study (Fig. 2A-B). As shown in Fig. 2C-E, all five candidate compounds significantly inhibited TG accumulation, decreased the number of lipid droplets and significantly downregulated the adipocyte differentiation marker genes *Pparg, Fasn, Fabp4 and C/ebpα* in OP cells. In addition, in the hepatic steatosis model, only E6446, not the four candidate compounds, suppressed the accumulation of TG content, the formation of lipid droplets and the expression of *C/ebpα*, *Fasn*, and *Fabp4* (Fig. 2F-H). In addition, none of the compounds affected the SCD2 expression, and E6446 had the strongest inhibitory effect on SCD1 activity (Figs. S1A-C; S12A-B). These results indicated that E6446 was the most potent inhibitor of adipogenic differentiation and hepatic lipogenesis among the selective compounds.

### E6446 targets SCD1 to inhibit adipogenic differentiation and hepatic lipogenesis

**Fig. 3:**
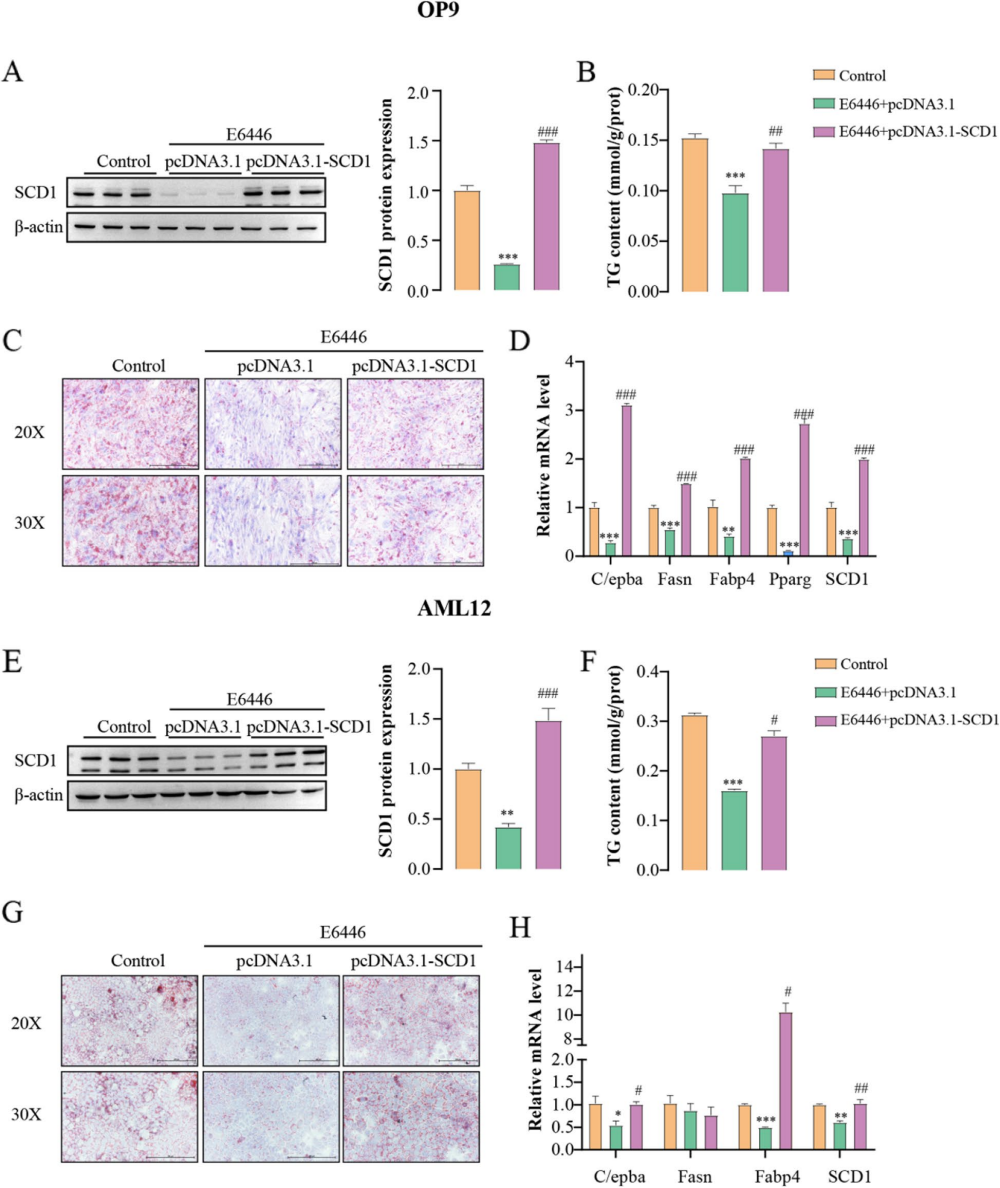
E6446 inhibits adipogenic differentiation and hepatic lipogenesis by targeting SCD1. OP9 and AML12 cells were transfected with the pcDNA3.1 vector or pcDNA3.1-SCD1. After transfection for 24 h, OP9 cells were incubated with 1 μM rosiglitazone for 15 days to induce adipogenic differentiation. Effect of E6446 (10 μM) on **A** SCD1 protein expression, **B** Oil Red O staining (scale bar: 200 μm, 250 μm), **C** cellular TG level, and **D** mRNA expression of adipogenic differentiation -related genes. After transfection for 24 h, AML12 cells were treated with a combination of palmitic and oleic acid (PAOA) for 48 h. Effect of E6446 (10 μM) on **E** SCD1 protein expression, **F** Oil Red O staining (scale bar: 200 μm, 250 μm), **G** cellular TG level, and **H** mRNA expression of lipogenesis-related genes. The values presented are the means ± SEMs of three independent experiments. *P < 0.05, **P < 0.01, ***P < 0.001 vs. the Control group. ^#^*P* < 0.05, ^##^*P* < 0.01, ^###^*P* < 0.001, vs. the E6446 + pcDNA3.1 group

Previous studies have found that E6446 can inhibit TLR7 and TLR9 signaling [22, 23]. However, TLR7 and TLR9 are primarily expressed in the brain and lymphoid tissue, and are barely expressed in the liver and adipose tissue. Our results are consistent with these findings, TLR7 and TLR9 was not detected in OP9 and AML12 cells (Fig. S2). To investigate whether E6446 targets SCD1 during adipogenic differentiation and hepatic lipogenesis, SCD1 was over-expressed and knockdown in OP9 and AML12 cells**.** The efficiency of transfection was shown in Fig. S3. The inhibitory effects of E6446 on adipogenic differentiation, including SCD1 protein expression, TG content and adipocyte differentiation marker gene expression were reversed when SCD1 was overexpressed in OP9 cells (Fig. 3A-D). In addition, E6446 also promoted the expression of UCP1 in OP9 cells (Fig. S4). Similarly, compared with the E6446 treatment group, SCD1 overexpression combined with E6446 treatment significantly increased SCD1 protein expression, lipid droplets, TG content and lipogenesis gene expression in AML12 cells (Fig. 3E-H). Meanwhile, knocking down SCD1 alone significantly inhibited both adipogenic differentiation and hepatic lipogenesis, and E6446 treatment did not enhance the phenotypes caused by SCD1 silencing alone (Fig. S5). Collectively, these results indicate that E6446 targets SCD1 to inhibit adipogenic differentiation and hepatic lipogenesis.

### E6446 is more potent than A939572 in preventing adipogenic differentiation and hepatic lipogenesis

**Fig. 4:**
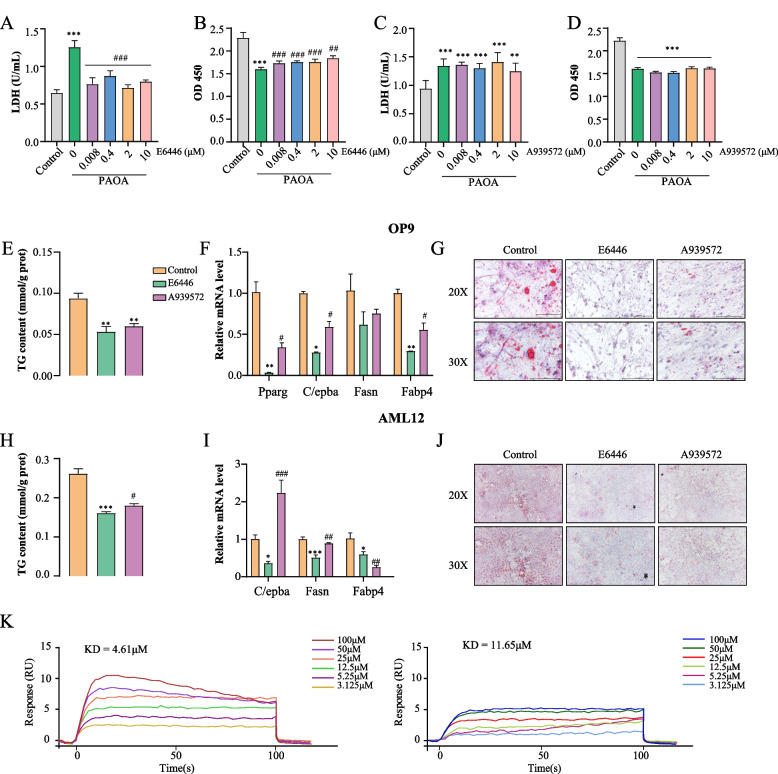
E6446 is more potent and safer than A939572 in preventing adipogenic differentiation and hepatic lipogenesis. The effect of different concentrations of E6446 and A939572 on LDH release and cellular viability in OP cells (**A**-**B**) and AML12 cells (**C**-**D**). For adipogenic differentiation, OP9 cells were incubated with 1 μM rosiglitazone for 15 days to induce adipogenic differentiation. The effect of E6446 and A939572 on **E** cellular TG level, **F** mRNA expression of adipogenic differentiation-related genes, and **G** Oil Red O staining (scale bar: 200 μm, 250 μm). During lipogenesis, AML12 cells were incubated with E6446 (10 μM) or A939572 (10 μM). The effect of E6446 and A939572 on **H** cellular TG level. **I** mRNA expression of lipogenesis-related genes, and (J) Oil Red O staining (scale bar: 200 μm, 250 μm). **K** Biacore X100 detected the kinetics/Affinity of E6446 and A939572 with SCD1. The values presented are the means ± SEMs of three independent experiments. *P < 0.05, **P < 0.01, ***P < 0.001 vs. the Control group. ^#^*P* < 0.05, ^##^*P* < 0.01, vs. E6446 group

Numerous studies have shown that A939572 is a potent SCD1 inhibitor, and many preclinical studies have used A939572 as a control (Stearoyl-CoA desaturase 1 is a novel molecular therapeutic target for clear cell renal cell carcinoma. Monounsaturated fatty acids generated via stearoyl CoA desaturase-1 are endogenous inhibitors of fatty acid amide hydrolase. Loss of stearoyl-CoA desaturase activity leads to free cholesterol synthesis through increased Xbp-1 splicing). The efficacy and safety of E6446 versus A939572 in the treatment of NAFLD is unclear. To address this question, we tried to reveal the advantages and disadvantages between them through a few simple classical experiments. LDH and CCK-8 assays indicated that E6446 significantly inhibited free fatty acid-induced lipotoxicity in hepatocytes while A939572 did not (Fig. 4A-D). In addition, E6446 and A939572 exhibited excellent SCD1 potency with IC50 values in the micromole range (E6446 IC50 = 0.98 μM; A939572 IC50 = 2.8 μM). Next, we investigated whether E6446 was more effective than A939572 in adipogenic differentiation and hepatic lipogenesis. As shown in Fig. 4E-G, we found that E6446 treatment was significantly better at decreasing the expression of the adipogenic differentiation marker genes *Pparg*, *Fasn*, *Fabp4* and *C/ebpα*, and lipid droplets than A939572 treatment. Moreover, compared to A939572 treatment, E6446 intervention significantly inhibited the accumulation of TG content, the expression of *Fasn*, *Fabp4* and *C/ebpα*, and the formation of lipid droplets (Fig. 4H-J). Using SPR assay, the kinetics/affinity of E6446 and A939572 with SCD1 were determined. The results showed that the K_D_ value of E6446 was 4.61 μM, while the K_D_ value of A939572 was 11.65 μM, indicating a strong interaction ability between E6446 and SCD1 (Fig. 4K). These results suggested that E6446 is safer and more effective than A939572 in NAFLD treatment.

### Transcriptomics analysis implicates ATF3 as a potential downstream target

**Fig. 5:**
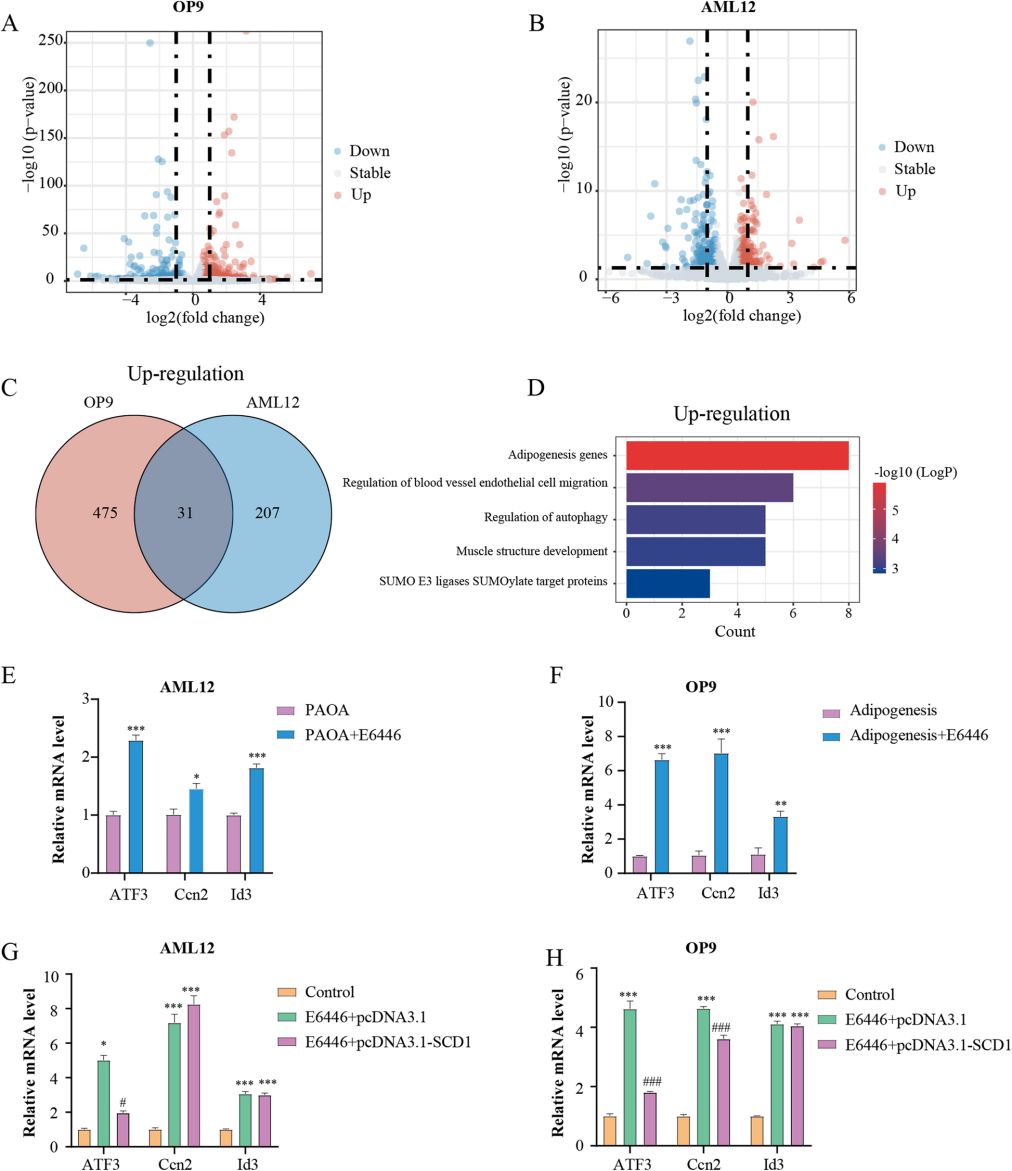
Transcriptomics implicates ATF3 as a potential downstream regulator. RNA-Seq was obtained from OP9 cells after 15 days and AML12 cells after 48 h with or without E6446 under model treatment. Volcano plot of the differentially expressed genes (DEGs) in OP9 (**A**) and AML12 cells (**B**). The Venn diagram (**C**) illustrates the common upregulated genes in OP9 and AML12 cells. **D** GO analysis of common upregulated genes in OP9 and AML12 cells. The top 3 genes with expression of adipogenesis gene terms in OP9 (**E**) and AML12 cells (**F**). **G**-**H** OP9 and AML12 cells were transfected with pcDNA3.1 vector or pcDNA3.1-SCD1. The relative mRNA levels of *Id3*, *Ccn2* and *ATF3* were measured with RT–qPCR after model establishment. The values presented are the means ± SEMs of three independent experiments. *P < 0.05, **P < 0.01, ***P < 0.001 vs. the Control group. ^#^*P* < 0.05, ^###^*P* < 0.001, vs. the E6446 + pcDNA3.1 group

To identify candidate molecules that might participate in E6446 mediated inhibition of adipogenic differentiation and lipogenesis, we performed RNA-seq in adipogenic differentiation and hepatic steatosis models after E6446 treatment. The analysis identified 506 upregulated and 167 downregulated genes in OP9 cells, and 238 upregulated and 167 downregulated genes in AML12 cells (Fig. 5A-B). Among the two upregulated gene sets, 31 genes overlapped (Fig. 5C). Subsequently, the co-upregulated genes were subjected to GO analysis, and the results indicated that the gene set was significantly enriched in adipogenesis genes (Fig. 5D). The top 3 genes among adipogenesis-related genes were *Id3*, *Ccn2* and *ATF3*. To verify the RNA-seq results, we performed RT–qPCR and found that *Id3*, *Ccn2* and *ATF3* were markedly increased during adipogenic differentiation and hepatic steatosis (Fig. 5E-F). However, when SCD1 was overexpressed, the *ATF3* mRNA level was significantly decreased in AML12 cells, while the *Id3* and *Ccn2* levels were not affected (Fig. 5G). Additionally, among the three genes, the *ATF3* mRNA level decreased most obviously in OP9 cells after SCD1 transfection (Fig. 5H). Therefore, these results indicated that ATF3 may be the potential downstream target of E6446-mediated SCD1 inhibition in adipogenic differentiation and hepatic lipogenesis.

### E6446 inhibited adipogenic differentiation and hepatic lipogenesis through SCD1-ATF3 signaling

**Fig. 6:**
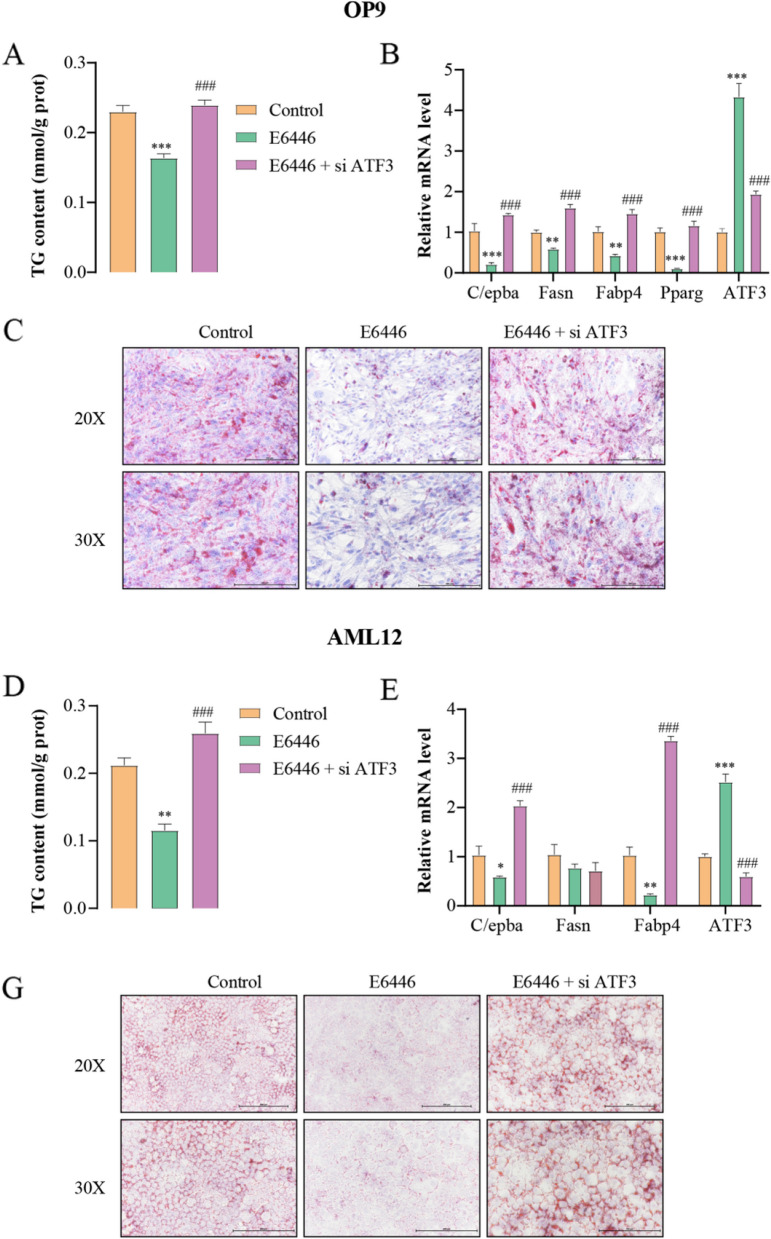
ATF3 silencing blocks E6446-induced inhibition of adipogenic differentiation and hepatic lipogenesis. OP9 cells were transfected with ATF3 siRNA or control siRNA and 24 h later, the cells were incubated with 1 μM rosiglitazone for 15 days to induce adipogenic differentiation. Effect of E6446 (10 μM) on **A** TG level, **B** mRNA expression of adipogenic differentiation-related genes, and **C** Oil Red O staining (scale bar: 200 μm, 250 μm). After transfection foe 24 h, AML12 cells were treated with a combination of palmitic and oleic acid (PAOA) for 48 h. Effect of E6446 (10 μM) on **D** TG level, **E** mRNA expression of lipogenesis-related genes, and **F** Oil Red O staining (scale bar: 200 μm, 250 μm). The values presented are the means ± SEMs of three independent experiments. *P < 0.05, **P < 0.01, ***P < 0.001 vs. the Control group. ^#^*P* < 0.05, ^##^*P* < 0.01, ^###^*P* < 0.001 vs. the E6446 group

The above results demonstrated that SCD1-ATF3 signaling might represent a key downstream signaling pathway for E6446 treatment. Thus, we investigated whether ATF3 silencing blocked the inhibitory effects of E6446 on adipogenic differentiation and hepatic lipogenesis. Our results revealed that ATF3 knockdown significantly prevented the E6446-induced inhibitory effects on adipogenic differentiation, including TG accumulation, adipocyte differentiation marker gene expression and lipid droplets formation (Fig. 6A-C). Similarly, ATF3 silencing also blocked the E6446 treatment-induced the inhibition of hepatic lipogenesis (Fig. 6D-F). These observations suggested that E6446 inhibited adipogenic differentiation and hepatic lipogenesis through SCD1-ATF3 signaling.

### E6446 improves HFD-induced NAFLD

**Fig. 7:**
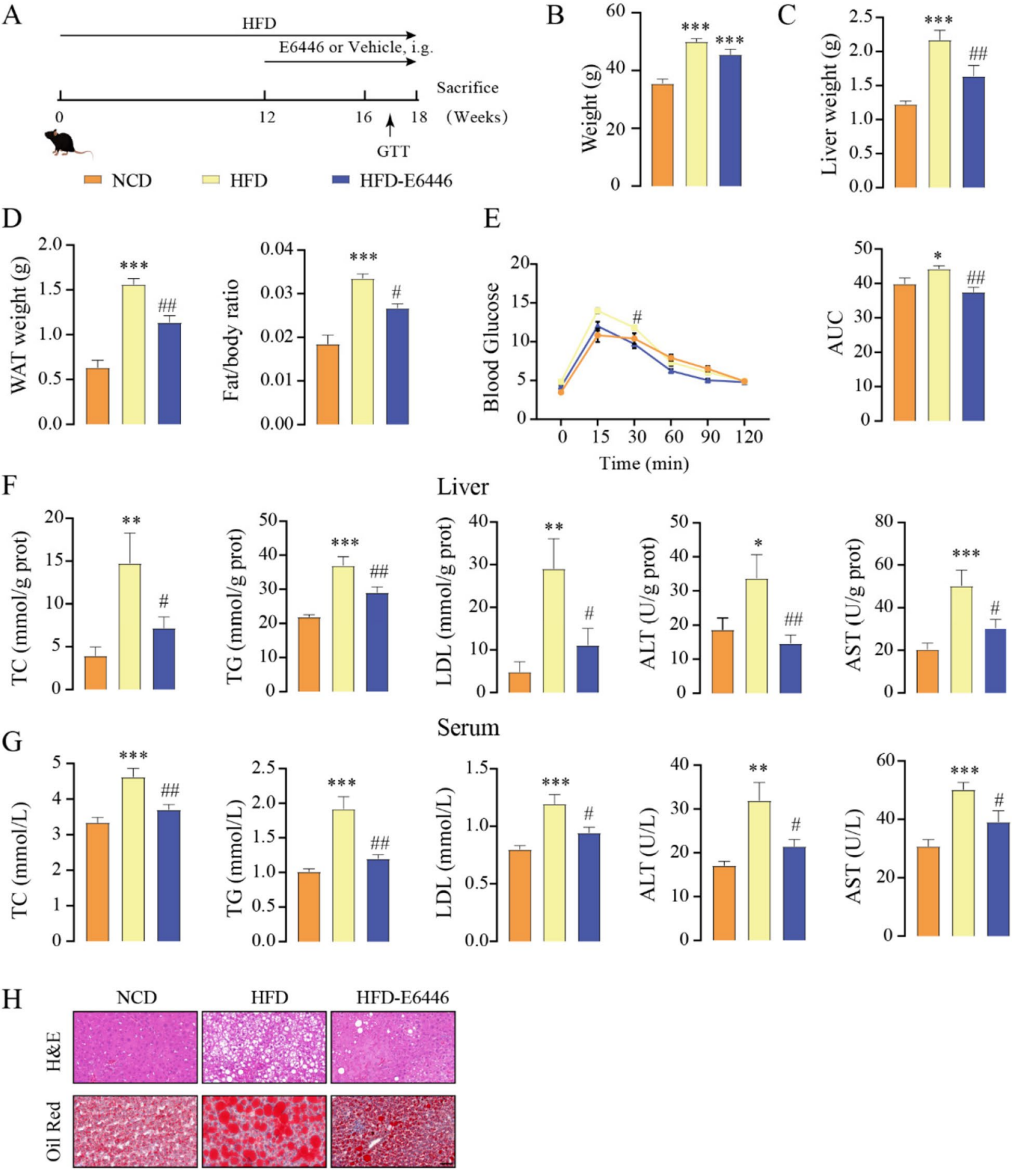
E6446 prevents HFD-induced NAFLD. C57BL/6J mice were fed a HFD for 12 weeks and supplemented with E6446 (20 mg/kg) or vehicle three times per week for 6 weeks. The mice were finally sacrificed, and further analyses were performed as indicated below. **A** Schematic of the experimental strategy. **B** Body weight. **C** Liver weight. **D** WAT weight and Fat/body ratio. **E** GTT. The hepatic (**F**) and serum (**G**) levels of TC, TG, LDL, ALT, and AST (*n* = 6). **H** Representative H&E (upper) and Oil red O (lower) staining of the liver sections of the mice in the indicated group. The values presented are the means ± SEMs. Scale bars, 50 μm. *P < 0.05, **P < 0.01, ***P < 0.001 vs. the normal control diet (NCD) group. ^#^*P* < 0.05, ^##^*P* < 0.01, vs. the HFD group

We next investigated the effect of E6446 on HFD-induced NAFLD in vivo. C57BL/6J mice were fed a HFD for 12 weeks and gavaged with E6446 or vehicle three times per week. HFD feeding for 18 weeks resulted in higher body weight, liver weight, and WAT weight than the normal control diet (NCD) group, while E6446 significantly reduced liver weight, WAT weight, and fat/body ratio (Fig. 7A-D). HFD-mice also showed more severe insulin resistance, as indicated by glucose tolerance tests (GTTs). E6446 significantly improved glucose tolerance (Fig. 7E). HFD-fed mice showed a significant increase in hepatic and serum TC, TG, LDL, ALT and AST compared the NCD group, while E6446 treatment significantly reduced these biochemical indices (Fig. 7F-G). H&E and Oil Red O staining revealed more lipid droplet in HFD-fed mice than in NCD mice, while E6446 treatment significantly decreased lipid droplets accumulation (Fig. 7H). These findings revealed that E6446 exhibited robust efficacy against HFD-induced hepatic steatosis.

## Discussion

**Fig. 8:**
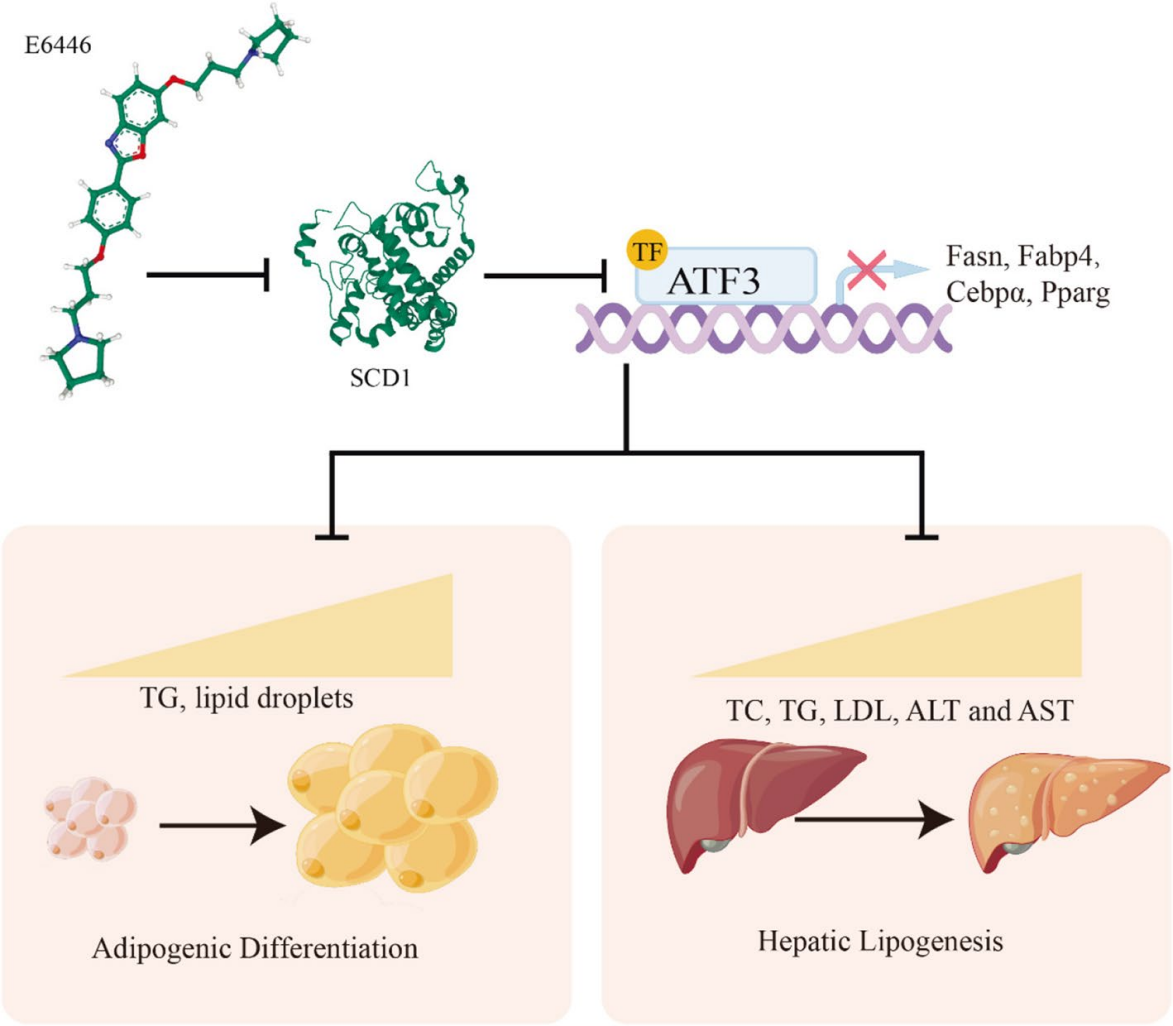
A proposed mechanism by which E6446 prevents nonalcoholic fatty liver disease (NAFLD). E6446 target the liver-adipose axis to suppress SCD1-ATF3 signaling, which mediates adipogenic differentiation and hepatic lipogenesis

The prevalence of nonalcoholic fatty liver disease (NAFLD) is rapidly increasing worldwide, and it poses a substantial social and economic burden worldwide. Despite significant efforts to explore the pathophysiological mechanisms underlying NAFLD, there are currently no drugs approved for NAFLD treatment. Drugs targeting the liver-adipose axis may exhibit potential in the treatment of NAFLD. Our study demonstrated that the novel SCD1 inhibitor E6446 suppressed two key processes of NAFLD development (hepatic lipogenesis and adipogenic differentiation) via SCD1-ATF3 signaling (Fig. 8).

From a clinical perspective, most SCD1 inhibitors are aimed at cancer treatment, and very few are aimed at NAFLD treatment. In our opinion, SCD1 is more valuable and meaningful as a therapeutic target for NAFLD than for cancer treatment. The most progressed clinical trial is aramchol, which has passed a clinical phase II trial and has shown promising efficacy in the treatment of NAFLD. The main reason for the therapeutic effects of aramchol may be, in part, that amramchol targets hepatic SCD1 [17, 24]. The degree of hepatic steatosis is determined by the amount of fat flux through liver cells [4, 25]. The flux of fat from adipose tissue to the liver is crucial. Importantly, the dysregulation of metabolic and immune factors in obese adipose tissue leads to the transport of fatty acids to the liver, promoting deposition of ectopic fat in the liver [26]. Lipotoxicity induces mitochondrial oxidative stress in the liver, further accelerating the progression of NAFLD [27]. In addition, some studies suggest that dysbiosis of the gut microbiota is also a contributing factor to the progression of NAFLD, the gut microbiota produces various metabolites that play a crucial role in regulating lipid metabolism in distant organs such as adipose tissue and the liver [28, 29]. However, SCD1 predominantly expressed in hepatocytes and adipocytes, and dysfunction of adipose differentiation is a critical driver of NAFLD and the liver-adipose cross talk that coordinates whole-body metabolism [30]. This cross-talk acts as a key player in modulating the initiation and development of NAFLD by regulating the flux of lipids and the production of cytokines and hormones that can affect hepatocyte function [31–33]. Targeting liver- adipose axis may be more efficacious than targeting liver or adipose alone in NAFLD. Therefore, there is an urgent need to develop SCD1-specific inhibitors that can target the liver-adipose axis. Based on high-throughput screening, we successfully identified a new specific SCD1 inhibitor, E6446, that suppressed adipogenic differentiation and lipogenesis which representant the two key processes between the liver and adipose tissue.

Activating transcription factor 3 (ATF3) is a member of the ATF/cAMP response element-binding protein family of transcription factors that regulates gene transcription by binding to the consensus sequence TGACGTCA in target gene promoters [34, 35]. Previous studies have shown that ATF3 can inhibit adipogenesis, induce adipocyte browning, prevent diet-induced steatohepatitis, and reverse steatohepatitis in db/db mice [36]. Mechanistically, ATF3 regulates adipogenic/lipogenic gene expression by binding to the C/EBPα, PPARγ, or ChREBP promoters [37, 38]. In this study, we found that E6446 suppressed the SCD1-ATF3 signaling, resulting in reduced adipogenic differentiation and lipogenesis. Similar to previous observations, SCD1 knockdown suppressed MYCN gene expression through ATF3 signaling pathways. These results suggest that SCD1 acts upstream of ATF3 signaling.

The earliest studies concluded that E6446 was a synthetic antagonist of nucleic acid–sensing TLRs, and it inhibited TLR7 and TLR9 signaling and inhibited the DNA-TLR9 interaction in vitro [39, 40]. However, TLR7 and TLR9 are mainly expressed in the brain and lymphoid tissue, and barely expressed in the liver and adipose tissue [41, 42]. In contrast, SCD1 is abundantly expressed in adipose tissue and the liver [11]. Our results were consistent with the above findings and indicated E6446 directly inhibited SCD1 rather than TLR7 and TLR9 in adipose tissue and the liver.

Our study was not without limitations. The HFD-induced NAFLD model does not fully represent the complex features of clinical NAFLD. Therefore, to evaluate the clinical feasibility of E6446 treatment for NAFLD, the effects of E6446 on different NAFLD models must be examined. Although SCD1 may act upstream of ATF3 signaling, as confirmed by our results, how SCD1 regulates ATF3 signaling has not been established.

## Conclusion

In summary, this study elucidates that the novel SCD1 inhibitor E6446 suppresses hepatic lipogenesis and adipogenic differentiation via SCD1-ATF3 signaling, providing insights into the role of E6446 in the treatment of NAFLD through the liver-adipose liver axis.

### Supplementary Information

**Additional file 1: Figure S1. **Effect of E6446 on SCD1 activity. The ratio of SCD1 product to substrate C16:1/C16:0 (A) and C18:1/C18:0 (B) in OP9 cells. Determination of C16:1 cellular fatty acid after adipogenic differentiation in AML12 cells (C). The values presented are the means ± SEMs of three independent experiments. *P < 0.05, ***P < 0.001 vs. the Control group.** Figure S2. **Effect of E6446 on SCD2, TLR7 and TLR9 expression. Effect of five potential compounds (10 μM) on SCD2 mRNA expression in OP9 (A) and AML12 (B) cells. (C) The mRNA level of TLR7 and TLR9 in mouse cortex, OP9 and AML12 cells. The values presented are the means ± SEMs of three independent experiments.** Figure S3.** The transfection efficiency in OP9 and AML12 cells. The transfection efficiency of si ATF3 (A) and pcDNA3.1-SCD1 (B) in OP9 cells. The effect of E6446 on exogenous SCD1 expression in (C) OP9 and (D) AML12 cells. The values presented are the means ± SEMs of three independent experiments. **P < 0.01, ***P < 0.001.** Figure S4.** Effect of E6446 on beigeing in OP9 cells. Quantification of UCP1, Prdm16, and Pgc-1a expression in OP9 cells. The values presented are the means ± SEMs of three independent experiments. **P < 0.01.** Figure S5. **SCD1 silencing blocks E6446-induced inhibition of adipogenic differentiation and hepatic lipogenesis. OP9 cells were transfected with SCD1 siRNA or control siRNA and 24 h later, the cells were incubated with 1 μM rosiglitazone for 15 days to induce adipogenic differentiation. Effect of E6446 (10 μM) on (A) TG level, (B) mRNA expression of adipogenic differentiation-related genes, and (C) Oil Red O staining (scale bar: 200 μm, 250 μm). After transfection foe 24h, AML12 cells were treated with a combination of palmitic and oleic acid (PAOA) for 48 h. Effect of E6446 (10 μM) on (D) TG level, (E) mRNA expression of lipogenesis-related genes, and (F) Oil Red O staining (scale bar: 200 μm, 250 μm). The values presented are the means ± SEMs of three independent experiments. *P < 0.05, **P < 0.01, ***P < 0.001 vs. the Control group.

## Data Availability

The datasets used and/or analyzed during the current study are available from the corresponding author on reasonable request.

## Abbreviations

ATF3: Activating transcription factor 3
ALT: Alanine aminotransferase
AST: Aspartate aminotransferase
AUC: Area under the curve
ECL: Enhanced chemiluminescent
FBS: Fetal bovine serum
FAME: Fatty acid methyl ester analysis
GO: Gene ontology
H&E: Hematoxylin and eosin
HDL: High-density lipoprotein cholesterol
HFD: High-fat diet
HRP: Horseradish peroxidase
GTT: Glucose tolerance testing
LDH: Lactate dehydrogenase
LDL: Low-density lipoprotein cholesterol
NGS: Next-generation sequencing
NAFLD: Nonalcoholic fatty liver disease
PDB: Protein data bank
PBS: Phosphate-buffered saline
SCD1: Stearoyl-coenzyme A desaturase
TC: Total cholesterol
TG: Triglyceride
