# Supplementary material for: Identification of novel SCD1 inhibitor alleviates nonalcoholic fatty liver disease: critical role of liver-adipose axis
Source: Cell Commun Signal. 2023 Sep 30;21:268. doi: 10.1186/s12964-023-01297-9 (PMC10544195; doi:10.1186/s12964-023-01297-9)

**Supplemental Material**

**Figure S1.** Effect of E6446 on SCD1 activity. The ratio of SCD1 product to substrate C16:1/C16:0 (A) and C18:1/C18:0 (B) in OP9 cells. Determination of C16:1 cellular fatty acid after adipogenic differentiation in AML12 cells (C). The values presented are the means ± SEMs of three independent experiments. *P < 0.05, ***P < 0.001 vs. the Control group.


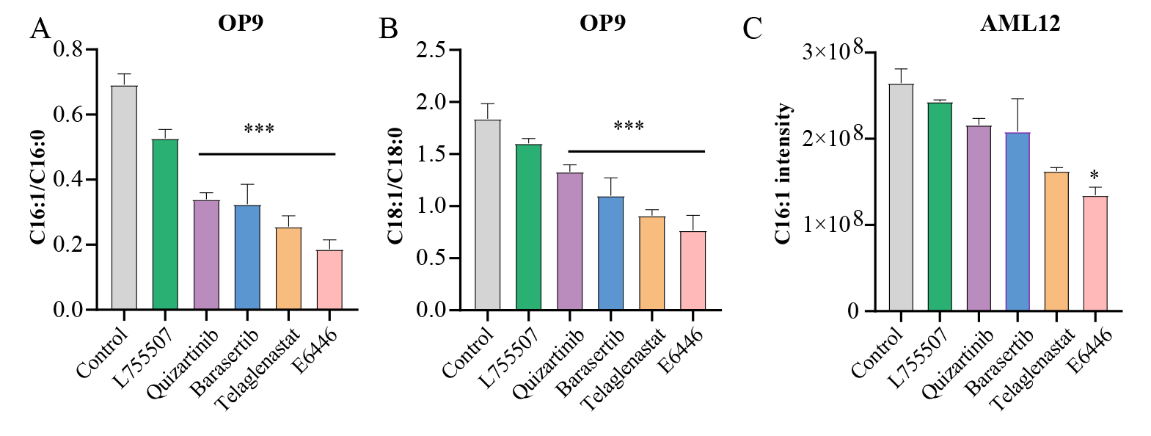


**Figure S2.** Effect of E6446 on SCD2, TLR7 and TLR9 expression. Effect of five potential compounds (10 μM) on SCD2 mRNA expression in OP9 (A) and AML12 (B) cells. (C) The mRNA level of TLR7 and TLR9 in mouse cortex, OP9 and AML12 cells. The values presented are the means ± SEMs of three independent experiments.


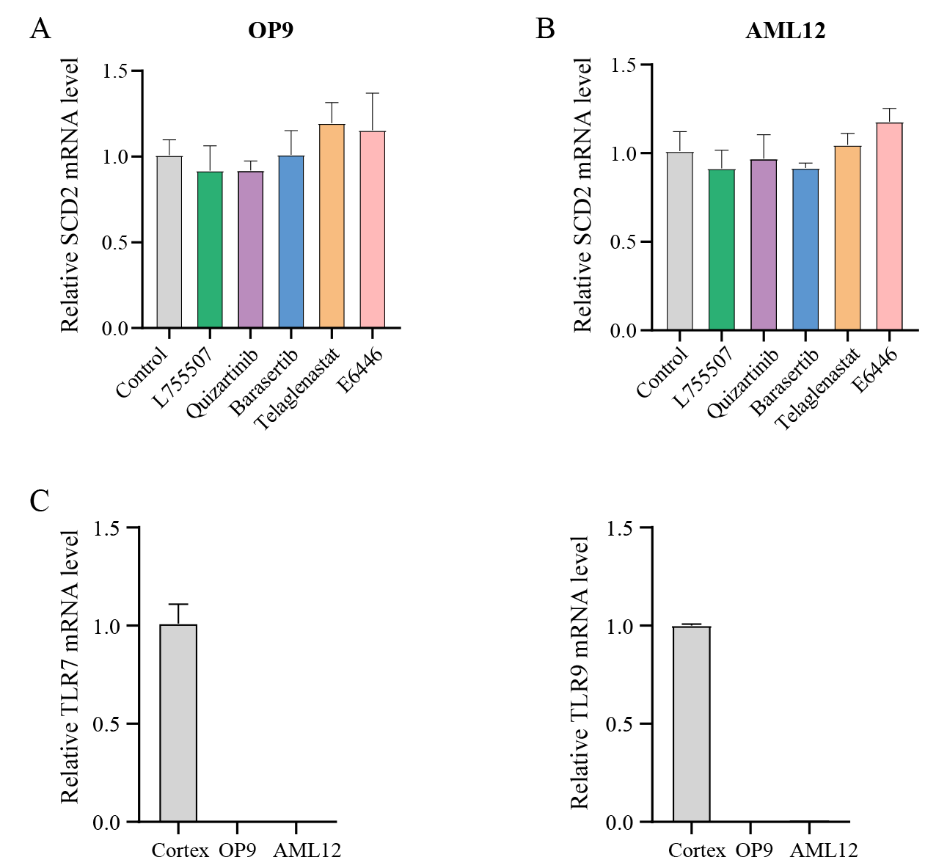


**Figure S3**. The [transfection efficiency](javascript:;) in OP9 and AML12 cells. The transfection efficiency of si ATF3 (A) and pcDNA3.1-SCD1 (B) in OP9 cells. The effect of E6446 on exogenous SCD1 expression in (C) OP9 and (D) AML12 cells. The values presented are the means ± SEMs of three independent experiments. **P < 0.01, ***P < 0.001.


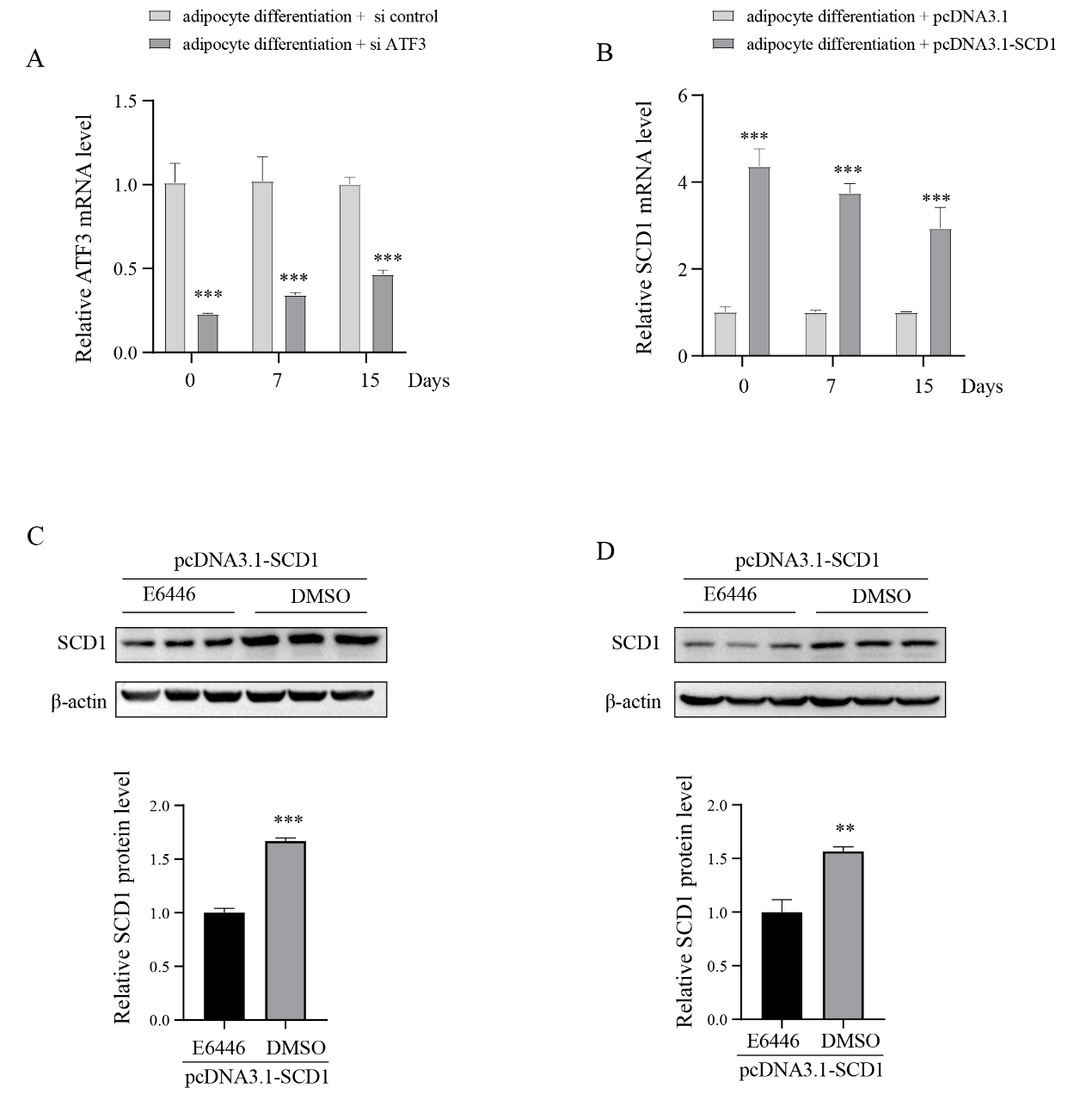


**Figure S4.** Effect of E6446 on beigeing in OP9 cells. Quantification of UCP1, Prdm16, and Pgc-1a expression in OP9 cells. The values presented are the means ± SEMs of three independent experiments. **P < 0.01.


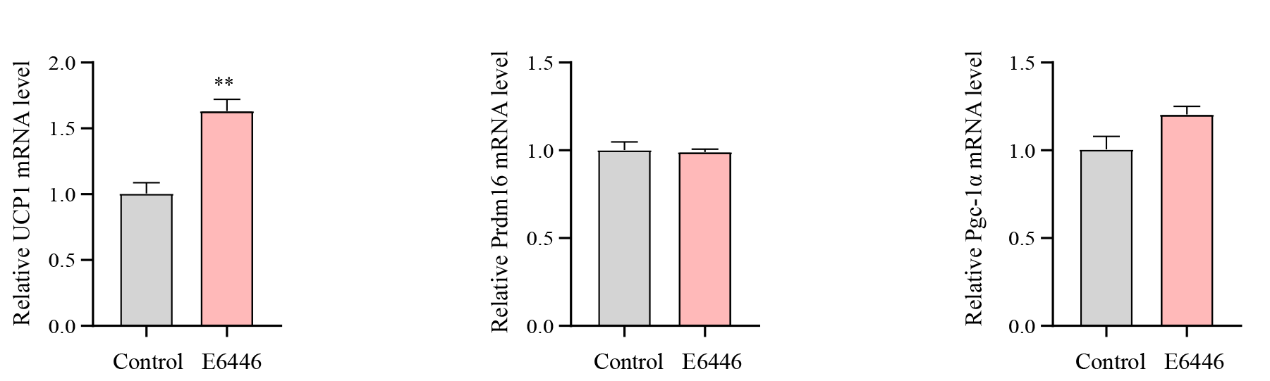


**Figure S5.**  SCD1 silencing blocks E6446-induced inhibition of adipogenic differentiation and hepatic lipogenesis. OP9 cells were transfected with SCD1 siRNA or control siRNA and 24 h later, the cells were incubated with 1 μM rosiglitazone for 15 days to induce adipogenic differentiation. Effect of E6446 (10 μM) on (A) TG level, (B) mRNA expression of adipogenic differentiation-related genes, and (C) Oil Red O staining (scale bar: 200 μm, 250 μm). After transfection foe 24h, AML12 cells were treated with a combination of palmitic and oleic acid (PAOA) for 48 h. Effect of E6446 (10 μM) on (D) TG level, (E) mRNA expression of lipogenesis-related genes, and (F) Oil Red O staining (scale bar: 200 μm, 250 μm). The values presented are the means ± SEMs of three independent experiments. *P < 0.05, **P < 0.01, ***P < 0.001 vs. the Control group.


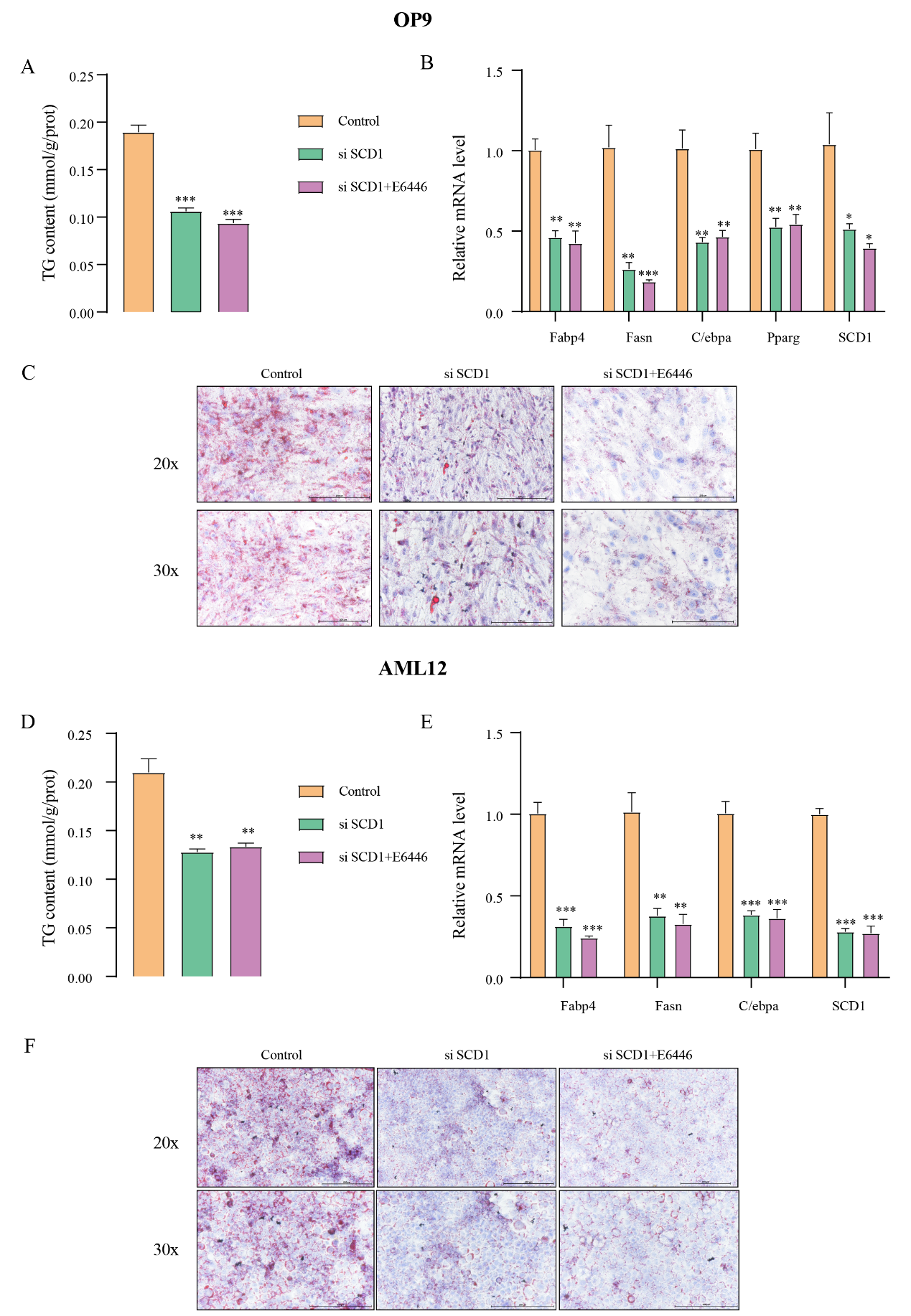

Supplement: Supplementary file 2 — Additional file 1: Figure S1. Effect of E6446 on SCD1 activity. The ratio of SCD1 product to substrate C16:1/C16:0 (A) and C18:1/C18:0 (B) in OP9 cells. Determination of C16:1 cellular fatty acid after adipogenic differentiation in AML12 cells (C). The values presented are the means ± SEMs of three independent experiments. *P < 0.05, ***P < 0.001 vs. the Control group. Figure S2. Effect of E6446 on SCD2, TLR7 and TLR9 expression. Effect of five potential compounds (10 μM) on SCD2 mRNA expression in OP9 (A) and AML12 (B) cells. (C) The mRNA level of TLR7 and TLR9 in mouse cortex, OP9 and AML12 cells. The values presented are the means ± SEMs of three independent experiments. Figure S3. The transfection efficiency in OP9 and AML12 cells. The transfection efficiency of si ATF3 (A) and pcDNA3.1-SCD1 (B) in OP9 cells. The effect of E6446 on exogenous SCD1 expression in (C) OP9 and (D) AML12 cells. The values presented are the means ± SEMs of three independent experiments. **P < 0.01, ***P < 0.001. Figure S4. Effect of E6446 on beigeing in OP9 cells. Quantification of UCP1, Prdm16, and Pgc-1a expression in OP9 cells. The values presented are the means ± SEMs of three independent experiments. **P < 0.01. Figure S5. SCD1 silencing blocks E6446-induced inhibition of adipogenic differentiation and hepatic lipogenesis. OP9 cells were transfected with SCD1 siRNA or control siRNA and 24 h later, the cells were incubated with 1 μM rosiglitazone for 15 days to induce adipogenic differentiation. Effect of E6446 (10 μM) on (A) TG level, (B) mRNA expression of adipogenic differentiation-related genes, and (C) Oil Red O staining (scale bar: 200 μm, 250 μm). After transfection foe 24h, AML12 cells were treated with a combination of palmitic and oleic acid (PAOA) for 48 h. Effect of E6446 (10 μM) on (D) TG level, (E) mRNA expression of lipogenesis-related genes, and (F) Oil Red O staining (scale bar: 200 μm, 250 μm). The values presented are the means ± SEMs of three independent experim [file 12964_2023_1297_MOESM1_ESM.docx]
